# Supplementary material for: Identification of Potentially Pathogenic Variants Associated with Recurrence in Medication-Related Osteonecrosis of the Jaw (MRONJ) Patients Using Whole-Exome Sequencing
Source: J Clin Med. 2022 Apr 12;11(8):2145. doi: 10.3390/jcm11082145 (PMC9030961; doi:10.3390/jcm11082145)
Supplement: Supplementary file 1 [file jcm-11-02145-s001.zip › Supplementary Figures S1 and S2.pdf]

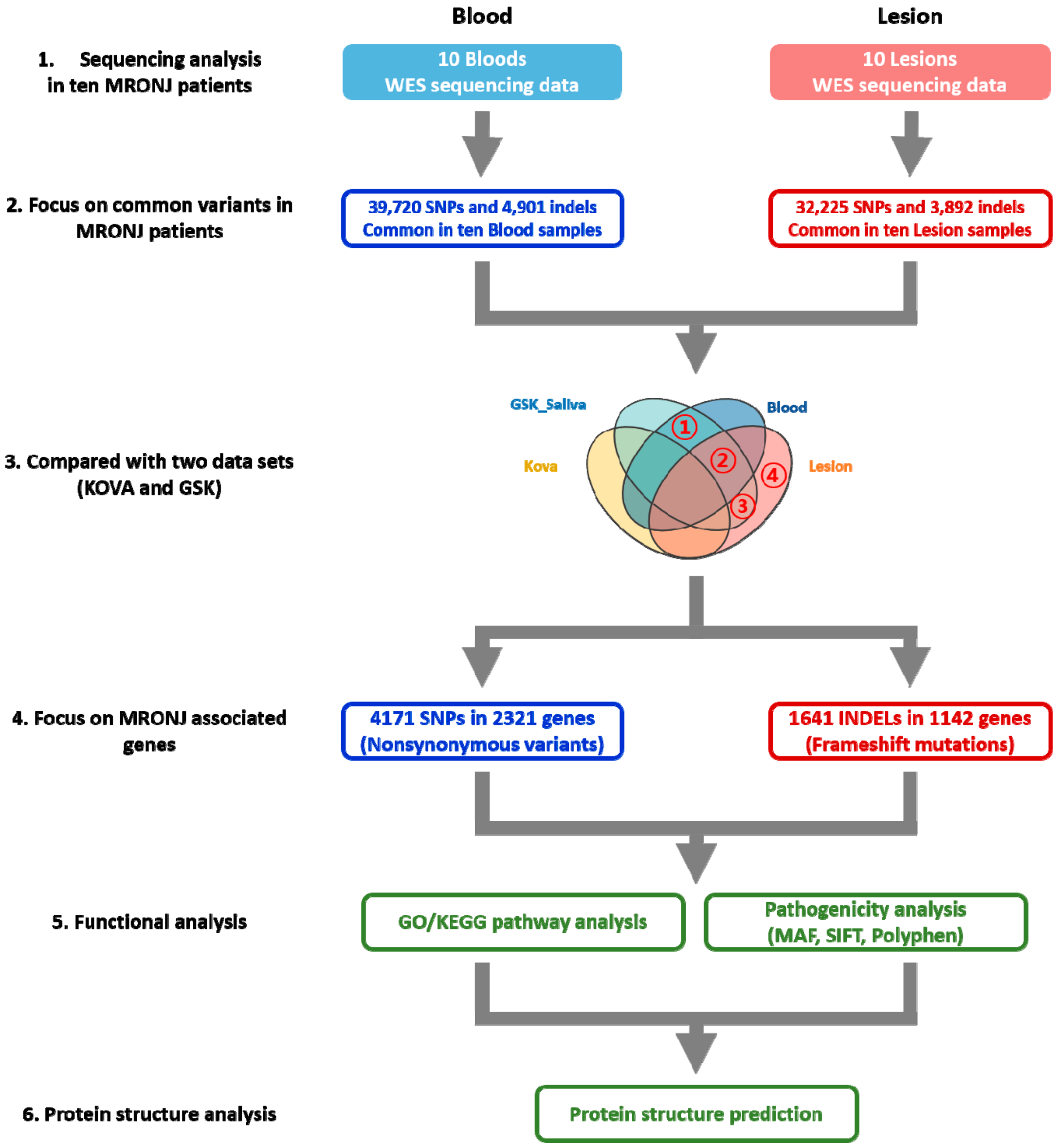

Figure S1: Outline of experimental design and analysis workflow for this study.

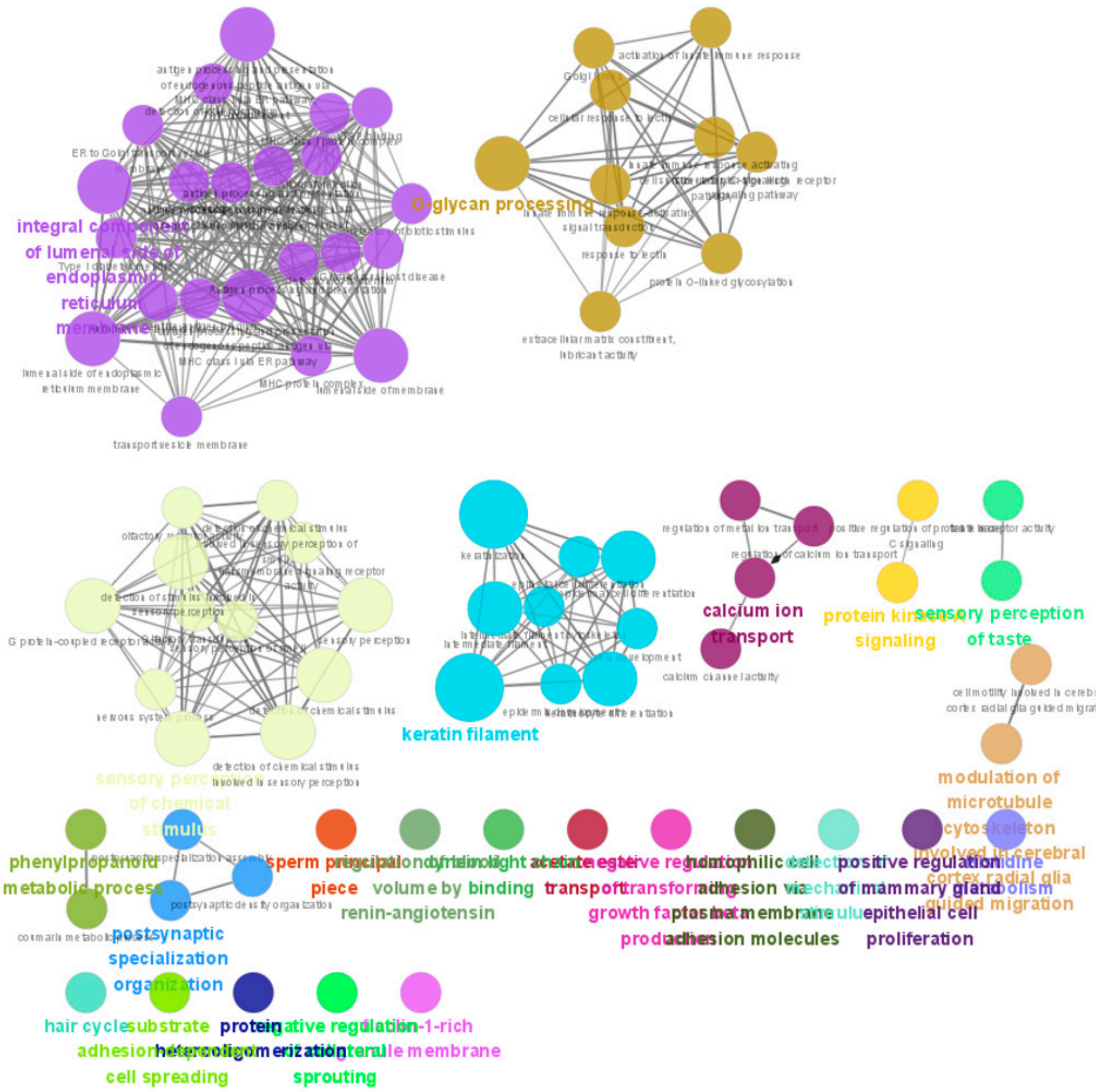

Figure S2: Molecular enrichment networks analysis of genes associated with MRONJ.
